# Supplementary material for: A Critical View of the Application of the APEX Software (Aqueous Photochemistry of Environmentally-Occurring Xenobiotics) to Predict Photoreaction Kinetics in Surface Freshwaters
Source: Molecules. 2019 Dec 18;25(1):9. doi: 10.3390/molecules25010009 (PMC7017383; doi:10.3390/molecules25010009)
Supplement: Supplementary file 1 [file molecules-25-00009-s001.zip › 3.2.4_gcc-4.4.0.zip!/3.2.4_gcc-4.4.0/3.2.4_gcc-4.4.0/doc/octave/html/interpreter/Inline-Functions.html]

Inline Functions - Untitled


Previous: Anonymous Functions,
Up: Function Handles Inline Functions and Anonymous Functions


---

#### 11.9.3 Inline Functions

An inline function is created from a string containing the function
body using the `inline` function. The following code defines the
function f(x) = x^2 + 2.

```
     f = inline("x^2 + 2");
```

After this it is possible to evaluate f at any x by
writing `f(x)`.

— Built-in Function: **inline** (str)  
— Built-in Function: **inline** (str, arg1, ...)  
— Built-in Function: **inline** (str, n)  
> Create an inline function from the character string str.
> If called with a single argument, the arguments of the generated
> function are extracted from the function itself. The generated
> function arguments will then be in alphabetical order. It should
> be noted that i, and j are ignored as arguments due to the
> ambiguity between their use as a variable or their use as an inbuilt
> constant. All arguments followed by a parenthesis are considered
> to be functions.
>
> If the second and subsequent arguments are character strings,
> they are the names of the arguments of the function.
>
> If the second argument is an integer n, the arguments are
> `"x"`, `"P1"`, ..., `"P`N`"`.
>
>
>
> **See also:** argnames, formula, vectorize.

— Built-in Function: **argnames** (fun)  
> Return a cell array of character strings containing the names of
> the arguments of the inline function fun.
>
>
>
> **See also:** inline, formula, vectorize.

— Built-in Function: **formula** (fun)  
> Return a character string representing the inline function fun.
> Note that `char (`fun`)` is equivalent to
> `formula (`fun`)`.
>
>
>
> **See also:** argnames, inline, vectorize.

— Built-in Function: **vectorize** (fun)  
> Create a vectorized version of the inline function fun
> by replacing all occurrences of `*`, `/`, etc., with
> `.*`, `./`, etc.

— Function File: **symvar** (s)  
> Identifies the argument names in the function defined by a string.
> Common constant names such as `pi`, `NaN`, `Inf`,
> `eps`, `i` or `j` are ignored. The arguments that are
> found are returned in a cell array of strings. If no variables are
> found then the returned cell array is empty.
